# Uptake of Soil-Residual Diazinon by Rotational Lettuce under Greenhouse Conditions

**DOI:** 10.3390/foods11213510

**Published:** 2022-11-04

**Authors:** Jihyun Yoon, Dajung Lim, Seungwon Lee, Jiyu Kim, Inseon Kim

**Affiliations:** Department of Agricultural Chemistry, College of Agriculture and Life Sciences, Chonnam National University, Gwangju 61186, Korea

**Keywords:** diazinon, lettuce, pesticide, positive list system, rotational crop, translocation, uptake

## Abstract

Pesticide residue is an increasing concern in rotational crop practices. The pesticide used for the primary crop may re-enter the secondary crop, thus exceeding pesticide levels set by the positive list system (PLS). As such, evaluation of pesticide residue translocated into rotational crops is required for ensuring pesticide safety. In this study, we investigated the residue pattern of diazinon translocated into lettuce as a typical rotational crop in Korea. Diazinon was used to treat greenhouse soil at the maximum annual application rate before crop planting. Diazinon residues in soil and lettuce were investigated using liquid chromatography/tandem mass spectroscopy and a modified quick, easy, cheap, effective, rugged, safe (QuEChERS) method. The limit of quantitation (LOQ) of diazinon was found as 0.005 mg/kg for the plant and soil samples. The recovery of diazinon at the LOQ and 10× the LOQ ranged from 100.2% to 108.7%. The matrix calibration curve showed linearity, with R^2^ values > 0.998. Diazinon residue in soil dissipated over time after the initial treatment, generating first-order kinetics (R^2^ = 0.9534) and having a half-life of about 22 days. The uptake ratio (UTR) of diazinon from the soil to the plant ranged from 0.002 to 0.026 over the harvest period. Considering the UTRs, diazinon residue in the edible leaf could exceed the PLS level (0.01 mg/kg) if lettuce is rotated in soil containing >0.357 mg/kg of diazinon. Based on our findings, to comply with the PLS, a 3-month plant-back interval is required following diazinon treatment and/or setting the maximum residue limit of diazinon for lettuce.

## 1. Introduction

Monitoring pesticide residue is especially important for fresh leafy vegetables, as they are widely consumed food items. A variety of vegetable crops are being used as greenhouse cultivation crops to meet the demand imposed by the growing human population [1]. In Korea, lettuce is cultivated as a typical rotational plant consumed throughout the year. The National Agricultural Products Quality Management Service of Korea has demonstrated that, among all crops on the market, lettuce ranks as the top crop in terms of the content of unregistered pesticides [2]. The unregistered pesticides in lettuce are transported to the plant from the soil after being used for the primary crop. Thus, managing pesticides remaining in soil is necessary to prevent pesticide contamination of secondary crops with unregistered pesticides. To ensure the safety of rotational crops, the positive list system (PLS), a pesticide safety system, was implemented in Korea for all agricultural products. Under PLS regulations, agricultural products containing unregistered pesticides for which the maximum residue limit (MRL) has not been established can be sold only when pesticides levels are <0.01 mg/kg. However, amounts in excess of this threshold may enter rotational crops through an unintentional route, i.e., even if the pesticides are not sprayed directly onto the secondary crops. In Korea, a cropping system in which different crops with short growing periods are rotationally cultivated in a greenhouse is commonly used; as such, pesticides used for primary crops could remain in the soil, thus contaminating secondary crops. For soil-residual pesticides used for primary crops that are not registered for use in secondary crops and remain at levels >0.01 mg/kg, products derived from the secondary crops cannot be sold under the PLS system. Thus, methods are required to prevent unintentional contamination with unregistered pesticides when cultivating secondary crops. To address this issue, the Rural Development Administration (RDA) of Korea requires the registration of pesticides for use with secondary crops, to decrease the possibility that soil-residual pesticides may contaminate secondary crops and violate the PLS. The RDA also applies the plant-back interval (PBI) as an alternative method for preventing unregistered pesticide contamination. However, in rotational cultivation systems, this requires information on the uptake and residue patterns of pesticides remaining in soil that have been used for primary crops, to avoid violation of the PLS.

Diazinon is an organophosphorus insecticide widely used for pest control in Korea. The World Health Organization (WHO) has categorized diazinon as a moderately hazardous pollutant of Class II [3]. The number of studies have demonstrated the toxicity of diazinon in aquatic organisms, including potential acute toxicity [4,5], body length shortening and endothelial cell changes [6], enzyme reaction and gene expression changes [7], lipid and protein oxidative damage [8] and adaptation adverse effects [9]. The reduction of intestinal microorganism, involving in the production of fatty acids, and the induction of bile acid disorder, by the exposure of diazinon have been reported in mice [10,11]. Moreover, the toxicological effects of diazinon on histophysiological and biochemical parameters in mammalian animals, particularly in the liver and kidney, suggest that diazinon residue cannot be ignored in life systems [12]. Taken from these studies, monitoring diazinon residue in food products has become increasingly important to ensure the safety of human health. According to the Institute of Public Health and Environment of Korea, diazinon ranks first among the top pesticides found in agricultural products on the market in the last 3 years [13]. Their report states that diazinon residue must be monitored continuously in leafy vegetable products, as it is mainly detected in vegetables in which diazinon is not registered for use. In this study, we aimed to investigate the magnitude of soil-residual diazinon translocated into lettuce as a representative rotational crop in Korea. The acceptable soil residue of diazinon when growing lettuce (<0.01 mg/kg) was estimated on the basis of the uptake ratio between the soil and crop. In turn, the PBI of diazinon was evaluated based on this threshold and the dissipation kinetics of diazinon in soil.

## 2. Materials and Methods

### 2.1. Chemicals and Reagents

Diazinon standard (98.2%) was purchased from Sigma-Aldrich Corp. (St. Louis, MO, USA) and its analytical standard (1000 mg/L) was obtained from Kemidas Corp. (Gunpo, Gyeonggi-do, Korea). Organic solvents used in this study were of HPLC grade, obtained from J. T. Baker (Phillipsburg, NJ, USA). Other chemicals were of analytical grade, obtained from Sigma-Aldrich Corp. (St. Louis, MO, USA), unless otherwise stated. Sample extraction and purification were performed using Agilent QuEChERS kits (San Francisco, CA, USA). A granular diazinon (3%) was kindly provided by Sungbo Chemicals Co., Ltd. (Seoul, Korea).

### 2.2. Diazionon Application

A granular formulation of diazinon (3%) was mixed thoroughly with soil (1:4 (*g/g*) in a stainless serum bottle and applied evenly onto each plot of soil at a rate of 50 kg/10a. The applied rate was determined based on the diazinon level used in greenhouse soil consecutively for five years. The soils of each plot were mixed by using a land management machine (Dongyang Tech., Daegu, Korea), as previously described [14].

### 2.3. Greenhouse Conditions and Experiments

The experiments were performed at an agricultural greenhouse (Damyang, Jeonnam, Korea). The soil composition was found as sand (50.4%), silt (37.6%) and clay (12.0%), 95.08 g kg^−1^ organic matter, 31.63 cmolc kg^−1^ cation exchange capacity and pH 6.8 by the method described previously [15]. The soil was investigated as a loam texture based on its composition data. The experimental plots were designed as prepared previously [5]. The plot size was 30 m^2^ with triplicates of each 10 m^2^. Lettuce was planted in the soil with the distance of 20 × 20 cm, 7 days after diazinon application. The plots were prepared to have a buffer zone (1.0 m) between each plot in order to prevent sample contamination. The greenhouse temperature and the humidity were monitored daily by a digital thermo-hydrometer during the experiment. The average temperature was 24.8 °C with the maximum 30.0 °C and the minimum 16.4 °C. The relative humidity was from about 48 to 88% during the experiments. The light was natural conditions during the experiment.

### 2.4. Sample Preparation

The soils were sampled by a stainless auger (Shinill Sci., INC., Paju, Korea) at a depth of 0–20 cm from eight points in each plot after diazinon treatment. The soil samples were dried overnight under dark shadow and subjected to pass through a 2 mm sieve. For the plant samples, lettuces (1 kg per plot) were collected from each plot 32, 36, 40, 44, 48, 51 and 56 days after treatment (DAT) with diazinon. The samples were put in polyethylene bags with ice and immediately transported to the laboratory. The plant root and leaf samples were prepared into small cakes after washed the debris with running water. The samples were then blended using a homogenizer and stored at −20 °C until used for the experiments.

For the determination of diazinon, the samples were prepared by methods modified from QuEChERS [16,17]. The methods were optimized to meet the requirements of the Organization for Economic Cooperation and Development (OECD) that has guided pesticide residue analysis. A 10 g soil sample was mixed with 10 mL distilled water in a 50 mL centrifuge tube for 15 min. The sample was then extracted with 10 mL of acetonitrile for 2 min and 4 g anhydrous MgSO_4_ and 1 g MgCl_2_ was added, followed by mixing thoroughly (2 min) and centrifugation at 3500 rpm (5 min). The supernatant (1 mL aliquot) was added with 150 mg MgSO_4_, 25 mg C18 and 25 mg PSA in a centrifuge tube and mixed for 2 min before centrifugation at 8000 rpm for 3 min. The supernatant was then subjected to passing through a 0.2 µm syringe membrane filter (PTFE-H) and used for liquid chromatography/tandem mass spectroscopy analysis (LC/MS/MS). For clean-up of the plant samples, the samples were prepared as described above, replacing C18 by graphitized carbon black (GCB, 2.5 mg).

### 2.5. Instrumentals

A Waters model Xevo TQ-XS triple quadrupole LC/MS/MS equipped with a Waters model ACQUITY^TM^ UPLC system was used for the sample analysis. The analytical column was a C18 stainless column (Osaka Soda CAPCELL CORE, 150 × 2.1 mm length, 2.7 μm particle size, 90 Å pore size). A solvent mixture of acetonitrile and water with 0.1% (*v/v*) formic acid was used as the mobile phase and flowed as: isocratic flow with 60% acetonitrile for 0.5 min, flow rate 0.3 mL min^−1^, gradient flow with 95% acetonitrile for 2.5 min, isocratic flow with 95% acetonitrile for 2 min. The collision energy values were 23 eV and 38 eV for quantitative and qualitative ions of diazinon, respectively. The electron spray ionization (ESI) method at positive ion mode was used for acquiring LC/MS/MS spectra with optimized conditions as follows: capillary voltage 3 KV, de-solvation N_2_ flow 650 L h^−1^, ion source temperature 150 °C, cone gas flow 50 L h^−1^, de-solvation temperature 350 °C and de-clustering potential value 31 eV. For optimizing LC/MS/MS, instrumental validation was conducted as guided by SANTE/11312/2021 [18]. The tolerances of ion ratio was permitted absolutely within ±30% by considering the relativity to the ratio of standard calibration. The diazinon MS ions were *m/z* 305.2 and *m/z* 169.1 for quantitative detection and *m/z* 305.2 and *m/z* 153.2 for qualitative detection, respectively. For quantitative analysis of diazinon in the samples, the matrix-matched calibration curve was applied in the range of 5 to 250 µg L^−1^ in the working solutions that had been diluted with the control extracts from the stock solutions (100 mg L^−1^). The limit of quantification (LOQ) at the signal-to-noise (S/N) ratio of 10:1 was calculated. The recovery tests at levels of LOQ and 10× the LOQ were conducted in triplicate.

## 3. Results

### 3.1. Validation and Establishment of Methods

For quantitative and qualitative analysis of diazinon, the methods used in the current study were established and validated based on the Organization of Economic Co-operation and Development (OECD) guidelines. The quick, easy, cheap, effective, rugged, safe (QuEChERS) method for sample extraction and clean-up was modified based on trial and error. Liquid chromatography/tandem mass spectrometry (LC-MS/MS) indicated high sensitivity and selectivity of the method (Table 1). Using established methods, standard calibration of diazinon achieved good linearity (0.005–0.25 mg L^−1^) for soil and plant samples. The coefficient of determination (R^2^) of the calibration linearity was 0.998–0.999 for all sample solutions, similar to neat organic solvent. The LOQ of diazinon in all samples was 0.005 mg kg^−1^. The matrix effects for the plant samples ranged from −8.21 to −7.10, while the matrix effect was 18.44 for the soil samples. The sample matrix generally affects quantitative measurements of pesticides when the matrix effect is >±10%. Thus, the matrix-matched standard calibration should be considered to improve method accuracy. In this study, we applied the matrix-matched standard calibration to all soil samples, given that the matrix effect was >±10%. The ion ratio tolerances in all samples (relative to the ratio for solvent standard calibration) ranged from −2.21 to 0.68, with ratios within ±30% of the guidelines for pesticide analysis set by the European Commission (SANTE/11312/2021) being considered acceptable [19]. Our method confirmed the presence of diazinon in all samples. Recovery tests were performed for samples with 1× and 10× the LOQ for diazinon to investigate method reliability. The recovery of diazinon ranged from 100.2% to 108.7% (Table 2). The relative standard deviation was <11.04%. These results met the guidelines of SANTE/11312/2021, thus validating our approach for determining diazinon levels in soil samples.

### 3.2. Residue and Uptake Patterns of Diazinon

**Figure 1:**
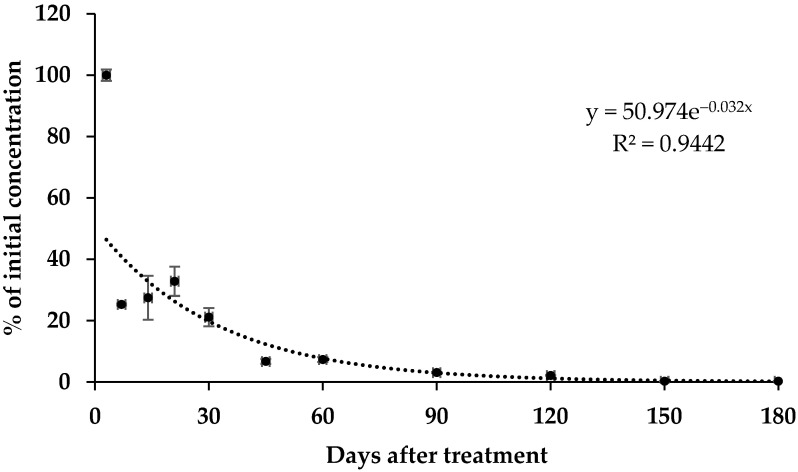
The pattern of diazinon dissipation in soil. Data are means ± standard deviation (SD) of triplicate.

Diazinon residue in soil was investigated over time to determine plant uptake patterns. Soil samples were obtained on each sampling day after treatment (DAT) and subjected to LC-MS/MS analyses. Diazinon residues decreased dramatically from the initial concentration to approximately 25% at 7 DAT, 7% at 45 DAT, and <1% at 180 DAT, as presented in Figure 1. The regression equation (C = 50.974e^−0.032t^; R^2^ = 0.9442) well described the first-order diazinon dissipation kinetics, where diazinon exhibited a half-life (DT_50_) of approximately 22 days. Diazinon appeared to dissipate rapidly after treatment, even when the level exceeded that recommended in Korea.

Diazinon residue was quantified in lettuce leaf and root over time; the residues ranged from 0.009 to 0.117 mg kg^−1^ in leaf samples and 0.250 to 1.573 mg kg^−1^ in root samples (Table 3). The residue levels in leaf and root samples appeared high at early harvest stage, 32 DAT and 36 DAT. The residue in the leaf sample decreased continuously from the initial concentration (0.093 mg kg^−1^) to 0.032, 0.024, and 0.009 mg kg^−1^ at 40, 48, and 56 DAT, respectively. The residue amount in the root samples was 1.020 mg kg^−1^ at 32 DAT, with a peak of 1.578 mg kg^−1^ occurring at 36 DAT; thus, the residue fluctuated over the growth period. The residue in the root samples on the last harvest day accounted for approximately 32.1% of that in the soil on that day, which suggests that diazinon from the soil was consistently absorbed by the roots over the course of the experiment.

The residual amounts of diazinon in leaf samples decreased continuously from 0.020 to 0.001 μg Plant^−1^ during the harvest period (Table 4), showing significant decrease after 36 DAT. A similar pattern was observed in root samples, decreasing significantly to 0.125 μg Plant^−1^ at 56 DAT from 4.437 μg Plant^−1^ at the initial harvest day (32 DAT). These results suggested that diazinon was diluted by plant growth.

The uptake pattern of diazinon was investigated based on the residue in the plant relative to soil. The uptake ratio (UTR) of diazinon was calculated as UTR = A × B^−1^, where *A* is the residue in plant parts and *B* is the residue in soil before planting. The UTR of diazinon over the growth period is presented in Table 5. The UTR in the leaf sample was 0.026 at 32 DAT, with a peak of 0.028 at 36 DAT followed by a steady decrease to 0.008 and 0.002 at 40 and 56 DAT, respectively (R^2^ = 0.8828). The UTRs of the root samples were higher than those of the leaf samples, ranging from 0.039 to 0.389. The UTR of the root samples peaked at 36 DAT; notably R^2^ = 0.3266 for the UTR, which was significantly lower than that of the leaf samples. The UTR of the root samples fluctuated during the harvest period, suggesting that roots take up diazinon continuously. The different UTR patterns between the leaf and root samples imply that diazinon accumulates in roots and is partially translocated into leaves.

### 3.3. Plant Back Interval of Diazinon

The PBI of diazinon was estimated to investigate when lettuce, as a secondary crop, may be safely planted in greenhouse soil containing diazinon. The highest UTRs among the calculated values were subjected to compare with the PLS level (0.01 mg kg^−1^) to determine the maximum soil residue (MSR), where MSR = 0.01 mg kg^−1^ × UTR^−1^. The MSR represents the diazinon residue transferable to lettuce at a concentration lower than the PLS level. The PLS level was applied to estimate the MSR, because the MRL of diazinon has not yet been set for lettuce in Korea. The MSR was then applied to the diazinon dissipation equation in soil (Figure 1), where MSR = 50.974e^−0.032T^; T refers to the PBI. The MSR value was 0.357 and 0.026 mg kg^−1^ for the leaf and root, respectively (Table 6). The uptake of diazinon generated a PBI of 93.9 and 177.7 days for the leaf and root, respectively. These results suggest that, when the primary crop is treated with diazinon, a >3-month interval after harvesting of the primary crop is necessary before lettuce can be cultivated in the same soil, given the MSR level. Our study suggest that guidelines for the safe use of diazinon in lettuce are required, given that lettuce is usually rotated into cultivation 7–14 days after the harvesting of primary crops in Korea.

## 4. Discussion

Lettuce is usually rotated into cultivation within a few weeks after primary crop cultivation in greenhouses in Korea. Lettuce is an important income crop for farmers because it is consumed throughout the year. However, unregistered pesticide levels in lettuce are among the highest for all crops. Unregistered pesticides may violate the PLS threshold for agricultural products; only crops with levels <0.01 mg/kg can be brought to market. Diazinon is one of the top 10 unregistered pesticides in crops on the market. It is mostly found in vegetables, such as lettuce and spinach. Thus, managing the residual diazinon in soil after its application to primary crops is required for safe rotational cultivation of vegetables in greenhouses. We studied the uptake of soil-residual diazinon in lettuce, as a typical rotational crop. LC-MS/MS methods were validated and developed for this purpose, in accordance with OECD guidelines. The methods are capable of quantitatively and qualitatively detecting diazinon without interfering with the samples, as evidenced by the matrix effects and ion ratio tolerance ratios.

Diazinon dissipated rapidly in soil after treatment, exhibiting a half-life of 22 days. Previous studies demonstrated rapid dissipation of diazinon in soil after treatment by microbial oxidation and hydrolysis [20,21,22,23]. Adsorption and volatilization are also important for the rapid dissipation of diazinon in agricultural soil [24,25]. Based on our data and the results of previous studies, the accumulation of diazinon in soil is not significant.

Diazinon residues in lettuce fluctuated during the harvest period, different from the residue patterns in soil. Diazinon uptake is likely greatest in the initial period after treatment, where the amounts in soil were low during the harvest. Diazinon uptake by carrot was high in the early growth stage [26], which is related to the high residue in soil in that stage. In this study, diazinon levels differed significantly between leaf and root, such that transport of diazinon from root to leaf may not be concentration-dependent. The peak UTR of diazinon in leaf was 0.028, while that of root was 0.389. These results suggest that the root took up diazinon continuously as the plant grew during the experiments. Moreover, diazinon dilution appeared to occur during plant growth, as in other studies [27,28]. The dilution effect was higher in leaf than root, probably due to leaf growth. Diazinon has a water solubility of 40 mg L^−1^ and octanol–water (log K_ow_) partition coefficient of 3.81 [29], indicating high sorption to soil [30]. There is a positive relationship between K_ow_ and the UTR [31]. High sorption of diazinon to soil could contribute to its rapid dissipation therein. Meanwhile, lettuce root hair would contact soil organics containing diazinon and affect the UTR. This hypothesis could explain approximately 32–42%, of the diazinon in soil, accumulated in lettuce root, relatively higher than the levels in lettuce leaf. Diazinon uptake by carrot was found higher on the root outer layer than the inside root body, meaning that direct contact of root with soil residue may contribute to the uptake [32]. The translocation of ^14^C-ring-labeled diazinon by bean plants documented the presence of diazinon only in the primary leaves by two days but not thereafter, persisting mostly in the roots [33]. Leaf vegetables such as lettuce spinach have been known to be a typical plant that takes pesticide moderately [34,35], but dissipation of pesticide is generally faster in lettuce than in other plants [36]. The residue behavior of methoxyfenozide and pymetrozine in Chinese cabbage was conducted for their risk assessment, demonstrating temperature-dependent dissipation, leading to low health risk [37]. The uptake of total soil-residual dinotefuran by lettuce has recently demonstrated that about a 24–28% level of initial concentration was translocated into leaves 30 and 60 days after treatment [38]. This high uptake ratio was explained by high water solubility and lower sorption to soil organics [39,40]. Thus, the evaluation of soil-residual pesticide in leaf vegetables is important for pesticide safety in agricultural products. In this study, diazinon in soil was taken up continuously by lettuce during the experiment, and the uptake was dependent on the soil-residual amount. Thus, managing diazinon residue in treated soil is necessary for safe rotation of lettuce in the greenhouse setting.

We aimed to identify an MSR resulting in lettuce diazinon uptake amounts <0.01 mg/kg, as stipulated by the PLS; the MSR satisfying this threshold was estimated as 0.357 mg kg^−1^ for lettuce leaf and 0.026 mg kg^−1^ for lettuce root. Considering the dissipation kinetics, a diazinon residence time in soil of 93.9 days for leaf and 177.7 days for root would be needed to achieve this MSR in soil; thus, a PBI of ≥3 months is needed to comply with the PLS. Efforts should also be made to reduce diazinon residue in soil to below the MSR before cultivating lettuce. An MRL for diazinon is required for lettuce. According to the OECD calculator [41], the MRL of diazinon for edible leaf parts was 0.3 mg kg^−1^. However, governmental input is needed for setting the MRL. According to the OECD guidelines [42], the PBI should be studied over a 7–30 day period for lettuce, given that it is a rotational crop. Following OECD guidelines, the RDA of Korea recommends conducting PBI studies over 30 and 60 days for lettuce and spinach, respectively [43,44]. Based on soil dissipation and UTR values for diazinon, we estimated a PBI of diazinon for lettuce complying with the PLS threshold.

The PLS was implemented in Korea in 2020 to promote safe pesticide use for all agricultural products intended for the market. However, the threshold of 0.01 mg kg^−1^ may be exceeded in rotational crops due to the uptake of residual pesticides in soil used for treating primary crops, as stated above. This may be especially common in Korea, where farmers cultivate a variety of rotational crops in greenhouses. Thus, studies on the uptake patterns of pesticides by secondary crops are needed to satisfy the PLS threshold. Here, we examined plant uptake of diazinon, as a typical unregistered pesticide that can accumulate in lettuce. Diazinon dissipated rapidly in the soil after treatment, and was taken up by lettuce over the growth period. To comply with the PLS threshold, a PBI of ≥3 months is needed for lettuce grown in greenhouse soil containing diazinon in an amount equivalent to the MSR. An MRL for diazinon should be set for lettuce grown in greenhouses, as an alternative to application of the PBI. Furthermore, a strategy for accelerating the dissipation of diazinon in soil [45] or suppressing diazinon uptake by the plant [46] would be helpful to satisfy the PLS threshold.

## 5. Conclusions

Diazinon in soil dissipated through first-order degradation kinetics and had a half-life of approximately 22 days. Diazinon was taken up continuously by lettuce during the growth period, and uptake was higher by root than leaf. The peak UTR was 0.028 for leaf and 0.389 for root. The UTRs were used to estimate the MSR that would prevent the PLS threshold (0.01 mg kg^−1^) for lettuce being exceeded; the estimated MSR was 0.357 mg kg^−1^ for leaf and 0.026 mg kg^−1^ for root. The PBI was calculated as 93.9 days for leaf and 177.7 days for root. Although diazinon dissipated rapidly in soil, continuous uptake throughout the harvest period suggests that soil-residual diazinon should be monitored carefully to comply with the PLS threshold, and we suggest a PBI of ≥3 months for rotational cultivation of lettuce in greenhouse soil. Methods to accelerate the degradation of diazinon in soil or decrease uptake by the plant would also be helpful, to ensure that lettuce planted after primary crops have been treated with diazinon is safe for consumption. An MRL of diazinon for lettuce should also be set.

## Figures and Tables

**Table 1:** Calibration equations, coefficient values of determinations, matrix effects, ion ratios and LOQ values of diazinon.

Matrix	Calibration Equation	R^2^	Matrix Effect (%) ^(1)^	Ion Ratio Tolerance (%) ^(2)^	LOQ (mg kg^−1^) ^(3)^
Soil	y = 16739x + 230.66	0.999	18.44	−2.21	0.005
Lettuce leaf	y = 8536x + 15.17	0.998	−7.10	−0.31	0.005
Lettuce root	y = 7826x + 286.32	0.999	−8.21	0.68	0.005

^(1)^ [(Slope in matrix-matched standard solution—slope in in solvent only)/(Slope in solvent only)] × 100%; ^(2)^ (Ion ratio in sample—ion ratio in solvent only)/(Ion ratio in solvent only) × 100; ^(3)^ Limit of quantification.

**Table 2:** Recovery of diazinon fortified in soil and plant samples.

Sample	Recovery (%) ^(1)^	
	LOQ ^(2)^	10× LOQ
Soil	104.2 ± 3.5	100.2 ± 3.5
Lettuce leaf	103.4 ± 11.4	103.3 ± 0.9
Lettuce root	108.7 ± 1.2	106.8 ± 0.6

^(1)^ Data are means ± SD of triplicate; ^(2)^ Limit of quantification.

**Table 3:** Diazinon residues in leaf and root samples during the harvest.

Harvest Numbers (DAT)	Residues (mg kg^−1^) *	
	Leaf	Root
1 (32)	0.093 ± 0.012 b	1.020 ± 0.033 b
2 (36)	0.117 ± 0.003 a	1.578 ± 0.0071 a
3 (40)	0.032 ± 0.017 c	0.250 ± 0.022 d
4 (44)	0.025 ± 0.004 cd	0.299 ± 0.030 d
5 (48)	0.024 ± 0.001 cd	0.159 ± 0.034 e
6 (52)	0.019 ± 0.001 de	0.387 ± 0.016 c
7 (56)	0.009 ± 0.001 e	0.418 ± 0.046 c

* Data are means ± SD of triplicate. Columns with the same letter are not significantly different by Duncan’s multiple range test (*p* < 0.05).

**Table 4:** Residual amounts of diazinon in lettuce during the harvest.

Harvest Number (DAT)	Diazinon Amount (μg Plant^−1^) *	
	Leaf	Root
1 (32)	0.020 ± 0.003 a	4.437 ± 0.143 a
2 (36)	0.011 ± 0.000 b	3.034 ± 0.137 b
3 (40)	0.001 ± 0.000 c	0.269 ± 0.024 c
4 (44)	0.001 ± 0.000 c	0.270 ± 0.027 c
5 (48)	0.001 ± 0.000 c	0.169 ± 0.037 c
6 (52)	0.001 ± 0.000 c	0.155 ± 0.007 c
7 (56)	0.001 ± 0.000 c	0.125 ± 0.014 c

* Data are means ± SD of triplicate. Columns with the same letter are not significantly different by Duncan’s multiple range test (*p* < 0.05).

**Table 5:** Uptake ratios (UTRs) of diazinon in lettuce.

Harvest Number (DAT)	UTRs *	
	Leaf	Root
1 (32)	0.026 b	0.252 b
2 (36)	0.028 a	0.389 a
3 (40)	0.008 c	0.062 d
4 (44)	0.005 cd	0.074 d
5 (48)	0.006 cd	0.039 e
6 (52)	0.004 de	0.095 c
7 (56)	0.002 e	0.103 c

* UTRs were obtained by dividing average residues in the plant parts by average residues in soil before planting. Columns with the same letter are not significantly different by Duncan’s multiple range test (*p* < 0.05).

**Table 6:** Plant-back intervals of diazinon for rotational cultivation of lettuce.

Plant Parts	PBI Factors			PBIs (Days) ^(4)^
	UTRs ^(1)^	PLS (mg kg^−1^) ^(2)^	MSRs (mg kg^−1^) ^(3)^	
Leaf	0.028	0.01	0.357	93.9
Root	0.389	0.01	0.026	177.7

^(1)^ The highest UTRs are used from Table 5; ^(2)^ positive list system level; ^(3)^ maximum soil residues taken by the plants lower than 0.01 mg kg^−1^; ^(4)^ plant-back intervals.

## Data Availability

All related data and methods are presented in this paper. Additional inquiries should be addressed to the corresponding author.
